# Accelerated NLRP3 inflammasome-inhibitory peptide design using a recurrent neural network model and molecular dynamics simulations

**DOI:** 10.1016/j.csbj.2023.09.038

**Published:** 2023-09-29

**Authors:** Bilal Ahmad, Asma Achek, Mariya Farooq, Sangdun Choi

**Affiliations:** a Department of Molecular Science and Technology, Ajou University, Suwon 16499, South Korea; b S&K Therapeutics, Ajou University, Campus Plaza 418, Worldcup-ro 199, Yeongtong-gu, Suwon 16502, South Korea; c Technology Development Platform, Institut Pasteur Korea, Seongnam 13488, Soouth Korea

**Keywords:** NLRP3, RNN, LSTM, Circular dichroism, Molecular dynamics simulation

## Abstract

Anomalous NLRP3 inflammasome responses have been linked to multiple health issues, including but not limited to atherosclerosis, diabetes, metabolic syndrome, cardiovascular disease, and neurodegenerative disease. Thus, targeting NLRP3 and modulating its associated immune response might be a promising strategy for developing new anti-inflammatory drugs. Herein, we report a computational method for *de novo* peptide design for targeting NLRP3 inflammasomes. The described method leverages a long-short-term memory (LSTM) network based on a recurrent neural network (RNN) to model a valuable latent space of molecules. The resulting classifiers are utilized to guide the selection of molecules generated by the model based on circular dichroism spectra and physicochemical features derived from high-throughput molecular dynamics simulations. Of the experimentally tested sequences, 60% of the peptides showed NLRP3-mediated inhibition of IL-1β and IL-18. One peptide displayed high potency against NLRP3-mediated IL-1β inhibition. However, NLRC4 and AIM2 inflammasome-mediated IL-1β secretion was uninterrupted by this peptide, demonstrating its selectivity toward the NLRP3 inflammasome. Overall, these results indicate that deep learning and molecular dynamics can accelerate the discovery of NLRP3 inhibitors with potent and selective activity.

## Graphical Abstract

**Figure:**
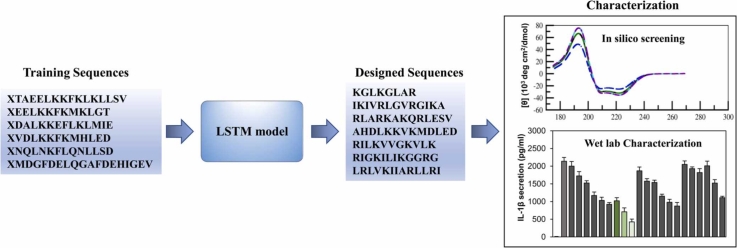

## 1 Introduction

The field of computational peptide design has recently undergone tremendous growth, with various advanced methods being developed to generate peptides with specific therapeutic properties [1]. These methods use artificial intelligence and machine learning to identify and generate candidate peptides. Some of these methods include DeepImmuno-GAN [2], codon-based genetic algorithm (CB-GA) [3] PepVAE [4], ProteinGAN [5], HydrAMP [6], PepGAN [7], and Peptide VAE [8], among others. However, one method that has received considerable attention and has been found particularly effective is the Long Short-Term Memory (LSTM) model [9], [10], [11]. LSTM models are well suited to predict the minimum inhibitory concentration (MIC) of peptides [12], which is a crucial factor in determining their therapeutic potency. This is attributed to the ability of LSTM to handle sequential data and capture long-term dependencies, which are critical features for peptide modeling [13]. Moreover, the success of LSTM models in natural language processing and time-series analysis underscores their potential in computational peptide design [14], [15].

The present study focuses on developing peptides targeting the NLRP3 inflammasome, a complex involved in the pathogenesis of diverse autoimmune and infectious diseases [16], [17]. The NLRP3 inflammasome is an attractive target for therapeutic intervention as it is activated by multiple stimuli and is associated with various health problems related to inflammation [18], [19]. Upon stimulation, NLRP3 oligomerizes through homotypic interactions between NACHT domains [19]. The oligomerized NLRP3 recruits ASC through homotypic NLRP3^PYD^ –ASC^PYD^ interactions and nucleates the formation of a helical ASC filament; this process is also mediated by ASC^PYD^–ASC^PYD^ interactions. The adaptor protein ASC bridges sensor proteins and caspase-1 to form ternary inflammasome complexes, assembled through PYD interactions between the sensors and ASC and through CARD interactions between ASC and caspase-1. ASC self-associates and binds NLRP3^PYD^ through equivalent protein regions, with a higher binding affinity for the latter [20]. The design of new therapeutic peptides is facilitated by examining structure-activity relationships [21], [22], [23], [24], [25], [26]; however, the complexity of the molecule space, intricate structure-function relationships, and multiple constraints present significant challenges. Artificial intelligence and statistical learning methods hold tremendous promise in overcoming these difficulties, as they can aid in predicting the properties of candidate molecules and streamlining the peptide design process [27], [28].

**Fig. 1:**
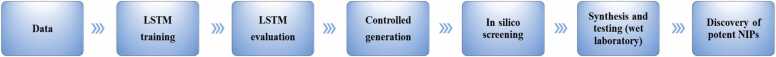
Approach overview. AI-driven approach to accelerate NLRP3-inhibitory sequence design.

In this study, we introduce a computational approach that integrates attribute-controlled deep generative models with physics-based simulations (depicted in Fig. 1).

This approach leverages the LSTM model for peptide generation and uses circular dichroism spectroscopic techniques to analyze secondary predictions. To gain a more comprehensive understanding of the properties exhibited by the generated peptides, we employed a combination of all-atom simulations and extensively analyzed their binding and physiochemical attributes. By leveraging the all-atom simulations, we could capture the fine details of peptide structures and explore their dynamic behaviors in a realistic molecular environment. Subsequently, through meticulous analysis of binding interactions and thorough examination of physiochemical properties, we obtained detailed insights into the binding affinity, stability, solubility, and other relevant characteristics of the peptides. The informed latent space produces novel and diverse peptides that are validated through extensive analysis, offering a promising solution for therapeutic peptide design.

## 2 Results

### 2.1 Design of peptides using the LSTM framework

**Fig. 2:**
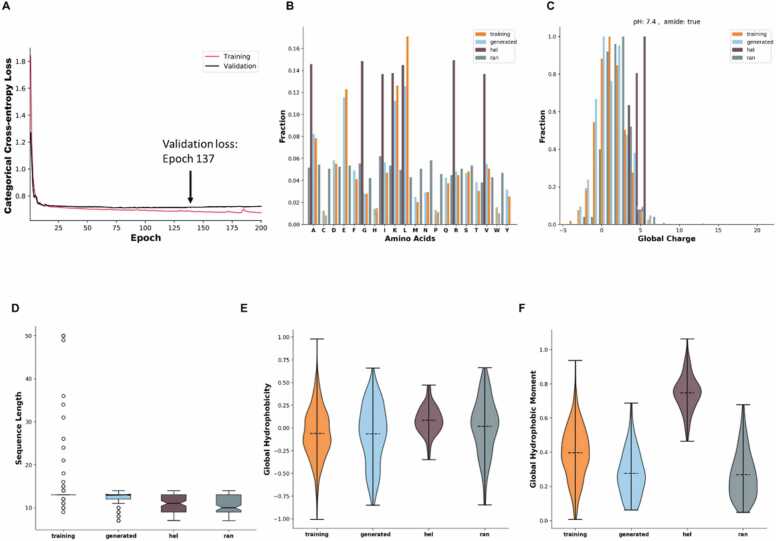
Comprehensive analysis of evolution loss during five-fold cross-validation and comparison of physicochemical properties (A) Categorical cross-entropy loss was computed for both training (red) and validation (black). Approximately 137 epochs are required to validate the average validation error. (B) Distribution of amino acids. (C) Global charge distribution. (D) Sequence length distribution of both the training data and generated sequences. The training data show a mean sequence length of 13.0 ± 2 and a median of 13, with minimum and maximum lengths of 9 and 50, respectively. Further, the generated sequences displayed a mean length of 12.6 ± 1 and a median of 13, with minimum and maximum lengths of 9 and 14, respectively. (E) Hydrophobicity, and (F) Hydrophobic moment. The bars show the generated peptides (generated, blue), random peptides (ran, grey), helical peptides (hel, brown), and original peptides (training, orange).

RNNs can recognize patterns in sequential data and generate new instances of data by capitalizing on context. We employed RNNs with LSTM units to recognize patterns in the sequential data of helical NLRP3 inhibitory peptides and their motif sequences. The trained models were used to generate de novo peptide sequences with properties similar to those of the original peptides. The best framework was used to generate *de novo* peptides. In this network, a categorical cross-entropy loss metric was utilized to evaluate the cross-validation and training outcomes (Fig. 2A). After conducting 137 validation epochs, the mean validation loss was determined to be 0.6 ± 0.06 (mean ± standard deviation). The value of 0.6 was deemed a crucial threshold, which represented an optimal balance between accuracy and generalization during the training process. It thus played a significant role in enabling the network to effectively learn from the data and make accurate predictions for unseen samples. This careful selection of the value 0.6 ensured that the network was trained in an effective and stable manner, resulting in robust and reliable outcomes on the entire dataset. We used the final state of the model to sample new sequences. Based on the consensus network model, 200 unique peptides were generated from 1018 sequences, which were not identical to the training data. Most of the newly generated sequences were between 9 and 14 residues in length (with a mean ± standard deviation of 12.6 ± 1.0), which aligns with the distribution of the training data endpoints. We then analyzed the main features of the generated sequences and compared them with the training data set in more detail (Fig. 2). The consistency between the generated and training sequences of observed min-max scaled feature values was evaluated using Welch's t-test, and the null hypothesis of equality was tested. The results of this analysis are presented in Table 1.

**Table 1:** Statistical analysis was conducted using Welch's t-test to compare the physicochemical properties of the generated sequences with those of random sequences.

Feature	Training	Generated	P-value
Charge	4.3 ± 3.1	3.4 ± 2.5	0.0249
Eisenberg hydrophobicity	0.85 ± 0.75	0. 74 ± 0.85	0.1918
Eisenberg hydrophobic moment	0.271 ± 0.14	0.298 ± 0.15	0.1897
length	13.0 ± 2.6	12.0 ± 2.6	0.0071
Isoelectric point	10.4 ± 2.0	9.3 ± 1.7	0.0001
Aromaticity	0.125 ± 0.11	0.110 ± 0.10	0.3142

The physicochemical properties highlighted in Table 1 were chosen because of their significant impact on peptide pharmacokinetic performance and biological activity. Peptide charge, Eisenberg hydrophobicity, hydrophobic moment, isoelectric point, and aromaticity play crucial roles in influencing various aspects of peptide behavior, such as membrane permeability, stability, proteolytic susceptibility, and binding affinity [29], [30]. Analyzing these properties provided valuable insights into the structural and functional characteristics of the generated NLRP3-inhibitory peptides.

The results of our analysis, presented in Table 1, further highlight the significance of these physicochemical properties. To assess the similarity between the generated sequences and the training data, we calculated the Euclidean distance in the combined descriptor space. The sampled sequences exhibited an average Euclidean distance of 0.9 ± 0.3 (mean ± SD). For comparison, we also computed the same distance for the random (1.1 ± 0.3) and helical (1.1 ± 0.3) sequence sets. The analysis demonstrated that our model-generated peptides exhibited a significantly higher similarity with the training data compared to that with the random and helical sequence sets (p-value < 0.05, Welch's t-test). This suggests that our model successfully generated peptides with characteristics resembling those present in the training data, enhancing their potential as modulators of NLRP3 inflammasome activation.

Overall, our focus on these physicochemical properties allowed us to optimize the molecular design of the peptides, leading to improved performance and effectiveness in targeting the NLRP3 inflammasomes. This offers promising prospects to develop potential therapeutic agents for inflammatory diseases.

### 2.2 In silico screening

**Fig. 3:**
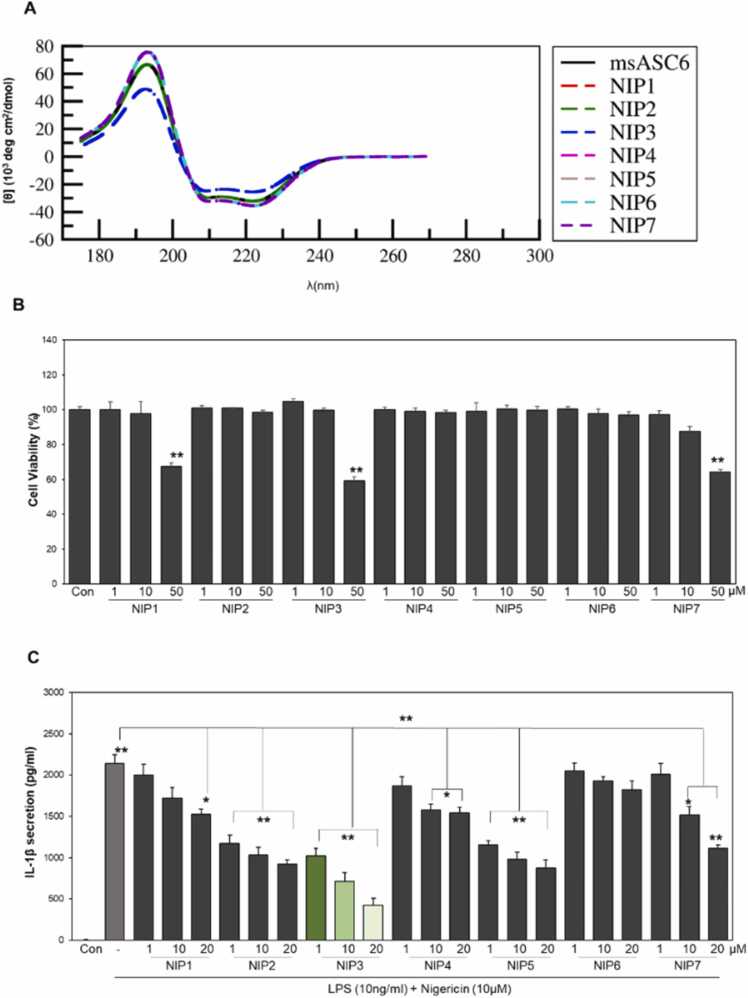
Comparative analyses of peptide properties and activities. (A) Comparison of circular dichroism spectra of selected peptides with msASC6 (B) Cell viability assay (MTT) after treatment with different concentrations of the designed NLRP3-inhibitory peptide (NIP). (C) The modulatory effect of these peptides on the NLPR3 signaling pathway was assessed using ELISA. Statistical significance was determined using t-tests, and multiple comparisons were corrected utilizing ANOVA. The presented data are representative of a minimum of four independent experiments (n ≥ 4); the bars indicate the means ± SD (*P < 0.05, **P < 0.01).

Generated sequences were quantified using a CD spectrum, binding affinity, hydrophobicity, and other physicochemical parameters (Fig. S1 and Table S1). Although CD spectroscopy is a highly sensitive technique for analyzing molecular structure, it provides limited resolution. While CD spectroscopy, on its own, may not provide high-resolution details about molecular structure, it can yield valuable insights when coupled with molecular modeling or complementary methods. This combined approach enhances our understanding of the molecular architecture under investigation. To address this, we employed SESCA [31] to compute electronic CD spectra of 3-D modeled peptides. The calculated CD spectrum and established peptides were compared (Fig. S1). In CD spectroscopy, a negative CD band at 220 nm typically signifies helical or strand structures, whereas a negative ellipticity peak at 195 nm often indicates disordered or denatured structures [32]. Peptides with ellipticity lower than that of the known peptides were removed. The selected peptides were then analyzed for physicochemical attributes, hydrophobicity, and helicity parameters (Fig. 3A and S2). The robustness of the SESCA method in discerning the CD spectra for determining protein secondary structure composition has been firmly established [33]. Employing an extensive reference protein dataset replete with high-quality CD spectra, deviations in CD spectra and reference structures attributed to experimental constraints were systemically assessed. Notably, this analysis identified intensity scaling errors and non-secondary structure contributions as the predominant sources of inaccuracies [34]. The investigation also highlighted how these inaccuracies can be mitigated through appropriate re-scaling and accounting for typical non-secondary structure contributions.

Leveraging the computational power of in silico simulations, we precisely assessed the binding strength of the peptides with the PYD. This efficient integration of in silico methods allowed us to explore and analyze several peptide sequences with a high degree of accuracy and without requiring laborious experimental procedures. Our approach enabled the efficient identification of promising NLRP3-inhibitory candidates, providing a solid foundation for further investigations and the potential development of innovative therapeutic agents targeting NLRP3 inflammasome activation. The optimized peptides were subjected to MD simulation and subsequent molecular mechanics Poisson-Boltzmann surface area (MMPBSA) analysis. This comprehensive approach enabled a thorough assessment of their interactions and binding affinity with the PYD molecule. The simulation provided valuable molecular-level insights into the interactions between the peptides and PYD, helping to better understand their binding strength. MD simulation can effectively filter high-throughput peptide sequences using physics-based methods, owing to its computational efficiency. To address the absence of established methodologies for identifying NLRP3-inhibitory candidates via molecular simulations, we conducted control simulations of sequences with known inhibitory activity against NLRP3 and compared them with sequences lacking such activity. Based on our control runs, we found a correlation between NLRP3 inhibition and variations in contact between positively charged residues and PYD (NLRP3^PYD^ and ASC^PYD^). Differentiation between high potency NLRP3-inhibitory and non-inhibitory sequences was specifically achieved by calculating contact variance, as depicted in Fig. S3 and presented in Table S1. Accordingly, this property measures the robustness of the binding of a peptide sequence to a PYD. Consequently, the generated NLRP3-inhibitory peptides (NIPs) that passed the classifier screening were further filtered using a contact variance cut-off of 3 (Table S1). The mean provides a measure of central tendency, whereas variance captures the spread or dispersion of data points, indicating how much they deviate from the average value. The incorporation of variance analysis facilitates a more comprehensive understanding of the dataset and allows more accurate and nuanced interpretations. In the context of activity prediction, a higher variance could suggest greater diversity or variability in the distances/contact numbers, potentially indicating a more dynamic or active system. Variance complements the mean by offering insights into the dispersion and variability in the data. This feature quantifies the strong binding tendency of a peptide sequence with the protein or membrane from a physical perspective [35], [36]. In our peptide analysis, out of 127 peptides examined, only 57 peptides were found to have a contact variance value less than or equal to 3. To focus on the promising candidates, specific metrics were employed for further filtering. We utilized a variance cut-off of 3 and a binding energy threshold of 17 kcal/mol as the criteria for this selection. These metrics were chosen for their relevance to peptide-protein interactions and their potential impact on the binding affinity of the peptide with the target protein. Upon conducting a comprehensive analysis, we identified the top 20 peptides that fulfilled both the variance and binding energy criteria. However, the high synthetic cost associated with peptide synthesis limited our capacity to synthesize all 20 peptides. Consequently, we could afford the synthesis of only 7 peptides from this final subset. This stringent evaluation criterion was employed to guarantee the stability of molecular interactions and structural integrity. The resulting peptides provided a favorable starting point for further investigations aimed at creating innovative and advanced therapeutic agents.

### 2.3 Wet-laboratory characterization

#### 2.3.1 In vitro screening and evaluation of the inhibitory effects of NIPs

The computational framework used in this study was based on screening LSTM-generated NIP sequences for their contact variance to differentiate between NLRP3-inhibitory and non-inhibitory candidates, as described above. Supplementary Table S1 presents the simulated and physicochemical properties of the sequences that passed this screening. Finally, the peptides were selected based on physiochemical characteristics, contact variance, and binding energy with PYD of NLRP3 and ASC (Table S1). The selected peptides with these optimized properties were fused with a cell-penetrating peptide to increase their cell permeability. These sequences (named NIP1 to NIP7) were tested for NLRP3 inhibitory activity in wet laboratory experiments. Cytotoxicity of the peptides was evaluated using an MTT assay. THP1-derived macrophages were treated with NIPs at concentrations ranging from 1 to 50 µM. The cell viability assay showed that, except for NIP2 and NIP4–6, the peptides were all cytotoxic at 50 µM (Fig. 3). Based on these results, treatment concentrations up to 20 µM were assumed to be safe, and accordingly, further experiments were conducted within the range of 1–20 µM.

**Fig. 4:**
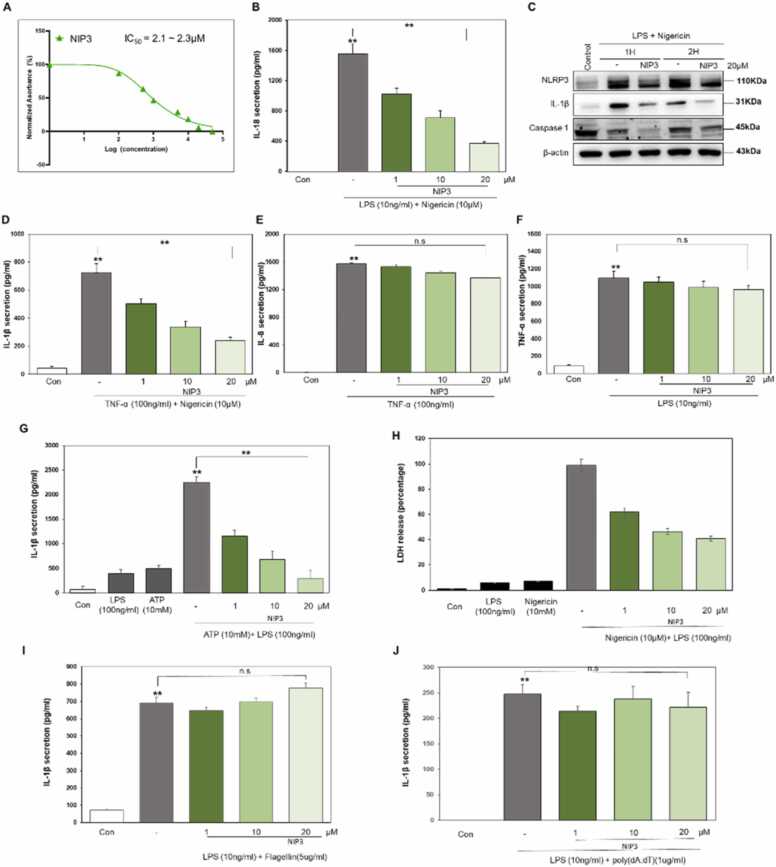
NIP3 suppresses NLRP3-mediated signaling in THP1-derived macrophages. (A) IC50 of NIP3. (B) The inhibitory impact of NIP3 was evaluated using an ELISA for secreted IL-18. (C) Expression levels of NLPR3, pro-IL-1β, and caspase-1 were quantified using western blot analysis of total protein extracts from THP1-derived macrophages treated with a concentration of 20 µM NIP3 peptide; β-actin served as a loading control. (D) NLPR3-mediated secretion of IL-1β was evaluated using an ELISA. (E, F) The effects of NIP3 on the TNFα-mediated (E) IL-8 secretion and LPS-mediated (F) TNF-α secretion were assessed using ELISA. (G) NIP3 attenuated ATP-induced IL-1β secretion, as quantified using ELISA in THP1-derived macrophages. (H) Cell death was determined using lactate dehydrogenase (LDH) release. (I-J) ELISA-based evaluation of IL-1β secretion mediated by (I) NLRC4 and (J) AIM2. Statistical significance was determined using t-tests, and multiple comparisons were corrected utilizing ANOVA. The presented data reflect the results of no less than four distinct experiments, with bars indicating the mean value ± SD (*P < 0.05, **P < 0.01).

NLRP3 inflammasome activation typically triggers the processing of pro-IL-1β and pro-IL-18, culminating in the mature forms of these cytokines and other inflammatory mediators being secreted. Therefore, NIPs were primarily evaluated for their modulatory influence on the NLPR3-mediated pathway via assessment of IL-1β secretion. THP1 macrophages were first primed with 10 ng/ml of LPS for 3 h. The cells were then treated with peptides at varying concentrations for 1 h. Next, the cells were incubated with 10 µM of nigericin for 2 h, and the supernatant was collected to evaluate IL-1β secretion. The experimental findings of our study demonstrate that NIP3 exhibits a greater inhibitory effect on NLPR3-mediated IL-1β secretion, with the extent of inhibition being dependent on the dose administered, as shown in Fig. 4C. To determine the IC50 value for NIP3, the peptide was tested in triplicate across a range of concentrations from 1 nM to 20 µM. This concentration range was carefully chosen to align with cytotoxicity data relevance, as demonstrated in Fig. S4. The cytotoxicity assay indicated a clear representation of the peptide effects on cellular viability, facilitating the precise determination of its IC_50_ value. The results of the study revealed IC50 values of 2.1–2.3 µM (Fig. 4A). Subsequently, the modulatory effect of this peptide on the NLPR3 pathway was studied by evaluating IL-18 secretion and evaluating the downstream protein expression levels of NLPR3 signaling. As expected, the amount of secreted IL-18 was significantly and dose-dependently decreased by treating the cells with NIP3 (Fig. 4B). A western blot analysis was performed to examine the effects of NIP3 on downstream proteins. The results showed that compared with untreated cells, those treated with NIP3 showed a notable decrease in NLPR3 expression, as well as reduced cleavage and activation of caspase-1 and IL-1β (Fig. 4C).

The analysis of the relationship between positive residues in AI-designed peptide sequences and their corresponding inhibitory potency, represented by IC_50_ values, is presented in Table S2. Positive residues, such as lysine (K) and arginine (R), play a crucial role in peptide-protein interactions owing to their positively charged side chains, facilitating important electrostatic interactions with negatively charged regions on the target protein. Upon data analysis, peptides with a higher count of positive residues evidently tend to exhibit enhanced inhibitory activity against the NLRP3, as indicated by lower IC_50_ values. For instance, NIP2, NIP3, and NIP5, which contain five, five, and three positive residues, respectively, display IC_50_ values of 10 µM, 2 µM, and 20 µM, indicating their potent inhibitory effects. Conversely, peptides with fewer positive residues, such as NIP1 and NIP4, exhibit IC_50_ values greater than 50 µM, suggesting relatively weaker inhibitory activity.

### 2.4 Inhibition of NLRP3-mediated cytokine production

Initiation of inflammasome assembly requires a two-step process of priming and activation. During the priming step, various pattern recognition receptors, such as TLR and NOD2, and cytokines, such as TNF-α and IL-1β, are upregulated. This results in activation of the transcription factor NF-κB, which subsequently triggers the transcription of genes responsible for detecting pathogen-associated molecular patterns and damage-associated molecular patterns [37], [38], [39]. The activation signal provided by these patterns, including LPS, launches multiple upstream signaling events, including potassium (K^+^) or chloride (Cl^−^) ion efflux, calcium (Ca^2+^) flux, lysosomal disruption, and mitochondrial dysfunction to produce reactive oxygen species, and triggers NLRP3 activation. Inflammasome formation activates caspase-1, which then cleaves pro-IL-1β and pro-IL-18 [19]. However, activation of NLRP3 using LPS follows a unique set of cellular events that differs from that of the canonical activation pathway.

Having identified NIP3 as a potent inhibitor of the NLRP3-mediated cytokine release and caspase-1 and IL-1β protein expression, we next investigated whether NIP3 affects NLRP3 inflammasome–proximate pathways. To rule out the possible influence of LPS as an NLRP3-independent stimulus, cells were instead primed with TNF-α. The data indicated that regardless of the priming signal used, NIP3 strongly inhibited the NLRP3 pathway (Fig. 4D). Furthermore, the impact of NIP3 on TNF-α secretion and TLR4-mediated signaling pathways was evaluated using the IL-8 and TNF-α secretion assays. The results showed that secretion of both cytokines was not significantly stalled, indicating the specificity of the new inhibitor for downstream signaling mediated by NLRP3 (Fig. 4E, F). To ascertain the specificity of the inhibitory effect associated with NLRP3 activation, we employed ATP as an alternative NLRP3 agonist to evaluate its influence on IL-1β protein secretion (Fig. 4G). Remarkably, the NIP3 peptide exhibited a discernible dose-dependent capability to attenuate IL-1β secretion prompted by ATP stimulation. This observation corroborates the distinctive inhibitory influence of NIP3 on NLRP3-mediated responses, highlighting its potential role as a modulator of the inflammasome-associated IL-1β response.

Assessment of pyroptosis, characterized by inflammatory cell death, involved quantifying the release of lactate dehydrogenase (LDH). Notably, the introduction of NIP3 (Fig. 3H) exerted a significant and compelling inhibitory effect on pyroptosis. This inhibition provided robust evidence for the direct dependence of cell death induced by LPS and nigericin treatment on NLRP3 inflammasome and caspase-1 activation. The NLRP3 inflammasome and caspase-1 play crucial roles in orchestrating the signaling cascade associated with pyroptotic cell death. These findings underscore the efficacy of NIP3 in modulating the NLRP3-caspase-1 axis and emphasize its potential in regulating pyroptotic cell death.

Furthermore, we investigated whether the NIP3 peptide can modulate other inflammasomes, such as NLRC4 and AIM2. The NIP3 peptide did not inhibit NLRC4-dependent IL-1β release in THP1 cells stimulated with flagellin and LPS (Fig. 4I). Further, IL-1β secretion was unaffected at varying concentrations of NIP3. Further, in our pursuit of comprehending the potential influence of NIP3 on AIM2-mediated IL-1β secretion, we employed LPS and poly(deoxyadenylic-thymidylic) acid [poly(dA:dT)] to activate AIM2 (Fig. 4J). The finding that NIP3 did not induce a notable reduction in IL-1β secretion in this context further accentuates the intrinsic specificity characterizing the inhibitory functionality of NIP3. This specificity strongly indicates that the regulatory effect of NIP3 distinctly targets the interface interaction between NLRP3 and ASC, thereby reinforcing the targeted nature of its modulatory action. Overall, these results indicate that NIP3 presents a discerning and precise inhibition mechanism targeting the activation of the NLRP3 inflammasome.

## 3 Discussion

Artificial intelligence research focuses on learning the implicit rules of interaction between complex molecular systems. One of the pressing requirements in scientific research is the development of novel molecules or materials that possess distinct structural and/or functional characteristics. Here, we consider NLRP3-inhibitory peptides as archetypical systems for molecular discovery. Dysregulation of NLRP3 inflammasome activation is a major causative factor for chronic inflammatory, metabolic, and neurodegenerative diseases as well as cancer. Pharmacological targeting of the inflammasome is thus being pursued as a strategy for treating auto-inflammatory diseases and cancer [40]. Owing to its complex signaling cascade, diverse targets can be used to inhibit NLRP3. Although a wide range of tactics to tackle the inflammasome have been employed for developing NLRP3 inhibitors, most of these have been designed to block the ATP-binding site. The shrimp-shaped structure of NLRP3 lacks an obvious binding pocket to which a drug can latch; therefore, finding drugs that selectively inhibit NLRP3 is challenging. The currently available pool of blockers against NLRP3–NLRP3, NLRP3–ASC, ASC–ASC, or ASC–caspase-1 interactions is less advanced [41]. Although targeting protein–protein interactions (PPIs) is challenging [42], this approach may be advantageous because it can facilitate a better approach and selective inhibition of NLRP3 inflammasome signaling. Therefore, instead of an agonist or antagonist, a signaling inhibitor (a biological therapeutic agent) with high specificity and greater potency can be chosen for drug development targeting NLRP3-NLRP3, NLRP3-ASC, or ASC–ASC PPI. However, we propose a computational framework that utilizes a controllable generative LSTM model, as depicted in Fig. 1, combined with physics-driven learning. This approach aims to design novel and effective NIP sequences from scratch and subsequently evaluate their efficacy and toxicity through experimental validation. Furthermore, the discovered peptide shows high efficacy against IL-1β and IL-18 in NLRP3-mediated pathways.

For inflammasome inhibition, to develop NLRP3 inhibitors that block NLRP3^PYD^–NLRP3^PYD^ and NLRP3^PYD^–ASC^PYD^ PPIs as well as suppress the activation of downstream signaling, we generated a library of peptides with the help of an RNN-based LSTM model trained on known NLRP3 peptides and motifs correlated with the PYDs of NLRP3 and ASC. Screening of the NLRP3 inhibitor library revealed NIP3 as a potential NLRP3 signaling inhibitor, which acts in a dose-dependent manner. Two other NLRP3 peptides, namely NIP2 and NIP5, were found to moderately suppress IL-1β and IL-18 (Fig. 2C). Our findings show that NIP6 and NIP7 were ineffective at inhibiting IL-1β and IL-18 secretion in a dose-dependent manner, as demonstrated in Fig. 2C. Notably, peptides with higher counts of positively charged residues (lysine and arginine) demonstrate enhanced inhibitory activity against NLRP3 inhibition, as indicated by lower IC_50_ values (potent inhibitory effects) (Table S2). Conversely, peptides with lower numbers of positively charged residues exhibit relatively weaker inhibitory activity, resulting in IC_50_ values greater than 50 µM. Positively charged residues play a crucial role in peptide-protein interactions, influencing the bioactivity of AI-designed peptides and their potential as rational design components for therapeutic agents targeting specific protein-protein interactions. Interestingly, the helicity of NIP6 and NIP7 is only slightly different from that of NIP3, as shown in Fig. S2. These results suggest that even minor variations in peptide structure may impact the ability to inhibit NLRP3 inflammasome activation. Further studies are thus needed to elucidate the precise structural features that contribute to the selective inhibitory effects of NIP3 on NLRP3 inflammasome activation.

Notably, NIP3 showed an IC_50_ of 2.1–2.3 µM (Fig. 4A), which was significantly higher than that of previously reported NLRP3 inhibitory peptides [21], [22]. Our binding-energy data revealed that NIP3 has a strong binding affinity for ASC^PYD^; further, this peptide also showed an affinity for NLRP3^PYD^. These findings are consistent with an NMR investigation that revealed the malleability of the NLRP3^PYD^ interface and the binding affinity of ASC^PYD^ for it [20]. Further, this region binds to NLRP3^PYD^, as previously reported [43]. In line with these observations, NIP3 may show similar affinity for several other proteins. Interestingly, we found that NIP3 inhibits the downstream signaling mediated by NLRP3 but not that mediated by IL-8 in the context of TLR4-mediated signaling (Fig. 4E, F).

NIP3 blocked NLRP3-mediated signaling and did not prevent the secretion of the cytokines TNF-α and IL-8 in the TLR4-mediated pathway in THP1-derived macrophages, indicating that this peptide specifically inhibits the IL-1β activation and IL-18 expression mediated by NLRP3, as evidenced by our western blot analysis (Fig. 4C). LDH measurements, as demonstrated in Fig. 4H, provided valuable insights into the role of NIP3 in regulating pyroptotic cell death, emphasizing its significance in modulating the NLRP3-caspase-1 axis. Furthermore, NLRC4- and AIM2 -mediated IL-1β secretion was uninterrupted by NIP3 (Fig. 4 I and J). These results led us to speculate that NIP3 specifically binds to the PYD domain of NLRP3 or ASC. An NMR study on ASC revealed the binding of ASC to both PYDs, indicating that ASC^PYD^ self-association and its interaction with NLRP3^PYD^ are mediated by almost identical binding regions [38]. NIP3-PYD binding alters intermolecular NLRP3-ASC oligomerization and ASC^PYD^ self-association and nucleation, thereby preventing the perpetuation of a massive inflammatory response. Either way, NLRP3 downstream signaling can be blocked by preventing NLRP3 oligomerization, its interaction with ASC through PYD, or both. Thus, the maturation of IL-1β and IL-18, as well as pyroptosis, are attenuated by inhibiting the NLRP3 inflammasome assembly. Consequently, NLRP3, which is involved in the pathogenesis of type 2 diabetes mellitus [44], atherosclerosis [45], cardiovascular [46], and neurodegenerative diseases [47], can be repressed by treatment with NIP3.

In recent times, several NLRP3 inflammasome pathway inhibitors have been documented, with select compounds demonstrating promising therapeutic capacity [41]. However, the small molecules often show off-target effects [48] as there is no obvious binding pocket for a drug to latch onto the shrimp-shaped NLRP3. Moreover, the NLRP3 inflammasome formation and cascade mechanism involves PPIs; therefore, the use of biologicals like peptides could be advantageous over that of small molecules. Inflammasome assembly is controlled by endogenous modulators of the pyrin or CARD domains, called POPs (PYD-only proteins) and COPs (CARD-only proteins). One study demonstrated that POP selectivity differed according to the peptide used [22]. POP1 and POP2 have been shown to possess broad inflammasome-inhibitory activity [43], [49], [50], [51], [52], [53]. However, POP3 specifically inhibits ALR inflammasomes [54]. In recent years, employing peptides for inhibiting PPIs has emerged as a promising approach to identifying bioactive compounds. NIP3 with IC_50_ in the 2.1–2.3 µM range is a promising therapeutic agent inhibiting the NLRP3 pathway. We thus believe that NIP3 can potentially alleviate inflammasome-associated diseases, as evidenced by our in vitro experiments. Further, our findings suggest that NIP3 holds great promise as a potential lead for developing peptidomimetics that can selectively inhibit NLRP3 inflammasome activation.

Numerous infectious and autoimmune diseases are attributed to abnormal intrinsic dysregulation of the NLRP3 inflammasome machinery [16]. In the present study, we discovered a peptide that effectively inhibits the NLRP3 signaling pathway. We demonstrated that the LSTM-based designed peptide, NIP3, exclusively targets the NLRP3 inflammasome axis, is effective in vitro, and significantly inhibits IL-1β and IL-18 from the NLRP3-mediated pathway. The results of this investigation illustrate the ability of the LSTM model and recurrent neural networks to produce unique amino acid sequences within the scope of the model and suggest their potential efficacy in the creation of proteins and peptides, obviating the need for exhaustive sequencing of libraries. In conclusion, this method has potential application to other protein-protein interaction systems beyond inflammasomes. However, extrapolating this method to other systems requires a deep understanding of the biology and biochemistry of the target protein and its interactions, as well as a thorough evaluation of the specific requirements for designing effective modulators. Furthermore, as each protein system is unique, different approaches may be required, necessitating tailoring the method to the specific needs of each system.

## 4 Materials and methods

### 4.1 Data collection and preprocessing

Formation of the NLRP3 inflammasome involves NLRP3, ASC, and caspase-1 and is characterized by a PYD-PYD interaction between NLRP3 and ASC (as shown in Fig. S5), as well as a CARD-CARD interaction between ASC and caspase-1 [55], [56]. Recent studies have identified peptides from inflammasome interaction domains [21], [22]. These studies have provided compelling evidence for the remarkable inhibitory effects of PYD target peptides on the NLRP3 inflammasome, effectively modulating their activity within the micromolar concentration range. To further explore these known NLRP3 peptides, we conducted a motif search using the inbuilt motif search program in MOE. The analysis performed in the study emphasized the potential application of BLAST-based and pattern-based sequences, with a specific emphasis on α-helical secondary structures, as a valuable approach for the rational design of innovative peptide therapeutics [57]. During training data selection, sequences with > 30% were selected to identify query sequences from the PDB database. The Cd hit [58] program was utilized to remove more than > 90% of sequence identity from the independent dataset. The final training set included 1008 peptides with lengths ranging from 9 to 15 amino acids, with an average sequence length of 12 residues.

### 4.2 Training of recurrent neural networks

**Fig. 5:**
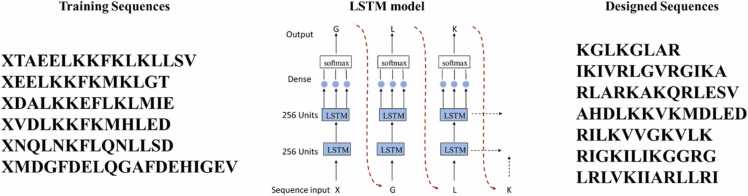
Schematic representation of the use of generative long short-term memory recurrent neural networks for de novo peptide generation. Training dataset sequences were padded with the token 'X' to match the length ‘n’ of the longest sequence string.

Recurrent neural networks (RNNs) are highly developed neural networks designed to manage sequence dependency. RNN models can identify patterns in sequential data and generate new instances of data based on the learned context. LSTM networks are a type of RNN used in deep learning and have proven successful in training very large architectures. These networks, which incorporate gate control units (input gate, forget gate, and output gate), are better equipped to learn the dependency between residues in peptide sequences compared to traditional RNNs [59], [60], [61]. Furthermore, they address the issue of gradients vanishing or exploding during the back-propagation stage in traditional RNNs. Several groups have used the LSTM or generative autoencoder architecture for de novo peptide design [8], [36], [62]. The LSTM network was used both for model training and peptide design. A diagram of the LSTM recurrent neural network architecture is shown in Fig. 5. In the LSTM network, the forget gate is responsible for determining which information to keep and which to discard as the network processes each element of the input sequence based on the previous input. It receives two inputs: new information (x_t) and the output of the preceding cell (h_t-1). It then filters the inputs with a sigmoid gate before merging them with the cell state by multiplication.

A type of recurrent neural network called a two-layer unidirectional LSTM RNN was utilized to generate peptide sequences without any prior knowledge of the amino acid sequence. The RNN comprised 256 memory units per layer; further, a densely connected feedforward layer with 22 output neurons was added after the second layer. The resulting output signals were combined using a SoftMax function to improve the accuracy of the model. Regularization of the LSTM layers in the first and second layers to prevent overfitting was achieved by using 30% and 45% dropout, respectively. According to Jozefowicz et al. [63], the LSTM forget gate used a fixed bias, and the Adam optimizer was tuned to a learning rate of 0.02. The categorical cross-entropy loss function (L) was applied to each hot-encoded residue present in a sequence comprising K amino acid symbols, as described by Equation (1). The calculation involved summing up the product of the target output (t) and the logarithm of the predicted output (y) for each residue (k) from 1 to K.

$$L\left(t,y\right)=-\sum_{k=1}^{K}t_{k}\log(\mathrm{yk})$$

Based on the training data, yk represents the predicted k-th one-hot vector from the Softmax layer, while tk represents the actual target k-th amino acid vector. Over 200 epochs, five-fold cross-validation was conducted with various model architectures, using 24, 48, 128, 256, and 512 LSTM cells in one or two layers to determine the optimal hyperparameters. In the regularization process, dropout was used with a fraction between 0.2 and 0.3 times the number of layers. The best-performing architecture was selected based on the validation loss observed at the minimum training epoch. To avoid the retention of residual knowledge from previous folds, the model weights were initialized with a new random seed for each cross-validation fold. In our analysis of de novo-generated sequences, we utilized two distinct criteria. First, we evaluated the validity and uniqueness of the sequences, analyzed global peptide descriptor values, and computed Euclidean distances in the combined descriptor space to assess their similarity with the training data. Second, we compared the generated peptides with two specific classes: "Random" sequences generated using randomization algorithms and "Helical" peptides designed to exhibit an amphipathic helical structure.

To generate these distinct peptide classes, we employed a sequence module [64], which allowed us to systematically explore diverse peptide designs. The "Random" sequences were generated de novo using randomization algorithms, whereas the "Helical" peptides were designed to possess amphipathic helical characteristics. Notably, all "random" and "helical" peptides were generated within our defined algorithms, and no external sources were involved in their selection.

This approach ensured the comprehensiveness of our peptide library, enabling us to thoroughly investigate and compare the structural characteristics of the generated peptides. By examining various peptide designs through these different criteria, valuable insights into their potential functionalities were obtained, enhancing our understanding of their performance in modulating NLRP3 inflammasome activation.

The neural network models were created using Python 3.7, TensorFlow 2.2, and the Keras high-level programming framework. Models were trained using Jupyter-Notebook on a workstation equipped with an Intel Xeon 1270 processor running NVIDIA GT 1070 graphics. The generated sequences were changed into a 3-dimensional structure using OmegaFold [65] and were subjected to evaluation for molecular properties. The molecular properties of the peptide were evaluated using SESCA (structure-based empirical spectrum calculation approach) [31]. We analyzed the CD spectra of the msASC6 peptide and compared it with those of other generated peptides. The msASC6 peptide, a 13-residue variation, was specifically designed to examine the impact of helical structures on the potency of inhibitory peptides targeting the NLRP3 inflammasome [66]. The results of the study showed that adding a staple sequence (I,I + 7) improved the helicity of the peptide, as measured using CD spectroscopy. Various physicochemical properties such as Eisenberg hydrophobicity, hydrophobic moment, aromaticity, charge, charge density, aliphatic index, hydrophobic ratio, isoelectric point, and instability index were determined using the global analysis method available in modLAMP [64].

### 4.3 Molecular docking, MD simulation, and binding energy calculation

Peptides optimized based on the aforementioned properties underwent molecular docking against the PYD of NLRP3 (PDB ID: 2NAQ) and ASC (PDB ID: 3J63) to evaluate their binding capabilities. Clusters with the highest population were examined to select representative solutions based on their docking score and root mean square deviation (RMSD) after clustering the docked solutions. Subsequently, the study employed MD simulations to obtain a thorough comprehension of protein-peptide interactions at an atomic level. MD simulations were conducted using GROMACS 2021.2 [67] on an Intel Xeon E5–2680 system with an Nvidia GeForce GTX 1070 graphics processing unit, as described in our previous research [68], [69].

Interacting residues between the peptide and PYD were identified using a threshold distance of 4.0 Å. Subsequently, we analyzed the computed averages and fluctuations of the interatomic distances of selected residues throughout the entire MD trajectories. Evaluation of the binding affinity of the optimized peptide with the PYD of NLRP3 and ASC involved the extraction of frames from MD trajectories and determination of the binding free energy by applying the molecular mechanics Poisson–Boltzmann surface area (MMPBSA) method, which has been previously described [70].

### 4.4 Peptide synthesis and cell culture

The peptides were selected based on ellipticity, helicity, charge, hydrophobicity, and isoelectric point and compared with the known NLRP3 peptides. To augment the intracellular delivery of selected peptides that met the predefined selection criteria, a strategic approach was employed. Specifically, these peptides were fused with a cell-penetrating peptide named penetratin (RQIKIWFQNRRMKWKK) at their N-terminus [71], [72], [73]. Penetratin can interact with the phospholipid bilayer, enabling its successful translocation across the cellular membrane and facilitating internalization into the cell. This fusion strategy capitalizes on the unique properties of penetratin to enhance the intracellular uptake of peptides, thereby promoting efficient delivery. The peptides were synthesized by Peptron (Ansan, Korea) with a high degree of purity (96% or more), which was verified using reversed-phase high-performance liquid chromatography on Shimadzu Prominence equipment. THP1-derived macrophages were used for in vitro peptide screening. Cells grown in the laboratory were sustained in RPMI 1640 medium supplemented with 1% penicillin/streptomycin solution and 10% fetal bovine serum (obtained from Thermo Fisher Scientific, Inc., Waltham, MA, USA). THP1 cells were induced to differentiate into macrophages by exposure to 80 nM of phorbol 12-myristate 13-acetate (Sigma-Aldrich Co., St. Louis, MO, USA) for 24 h. Subsequently, the cells were kept in a CO_2_ incubator at 37 °C with 5% humidity (Thermo Fisher Scientific, Inc.), and after 18 h of incubation, the media were replaced.

### 4.5 Cell viability assay

THP1 cells were seeded into the wells of a 96-well plate at a density of 10^5^ cells per well and then left overnight to grow undisturbed. Cell viability was then assessed using a colorimetric assay that employed 1-(4,5-dimethylthiazol-2-yl)− 3,5-diphenylformazan (MTT) reagent, following the manufacturer's instructions (Sigma-Aldrich Co.).

### 4.6 Enzyme-linked immunosorbent assay (ELISA) and cell treatment

THP1 macrophages were cultured in 96-well plates (BD Biosciences, Franklin Lakes, NJ, USA) at a density of 10^5^ cells per well and incubated overnight. The next day, the cells were primed with LPS (Sigma-Aldrich) at 10 ng/ml or with human tumor necrosis factor alpha (TNF-α; R&D Systems, Minneapolis, MN, USA) at 100 ng/ml for 3 h, followed by treatment with various peptide concentrations for 1 h. To activate the NLRP3 pathway, cells pre-primed with LPS were stimulated with 10 µM nigericin (Invivogen, San Diego, CA, USA), a known activator of NLRP3, for 2 h. After 6 h of treatment, IL-1β and IL-18 secretion levels were assessed using the human IL-1β Uncoated ELISA Kit (Invitrogen, Waltham, MA, USA) and the human IL-18 ELISA Kit (RayBiotech, Norcross, GA, USA), respectively. The human IL-8 and TNF-α ELISA Kit (Invitrogen) was used to measure the quantities of IL-8 and TNF-α, respectively. In a distinct experimental approach aiming to evaluate the specificity of the NIP3 peptide in the context of NLRP3 inflammasome modulation, THP1 cells were cultured and meticulously seeded at a density of 5 × 10^4^ cells per well in a 384-well plate after PMA-induced differentiation. After careful cell seeding, the experimental design was as follows: an initial priming phase lasting 4 h involved the application of LPS (10 ng/ml); this was followed by a subsequent 1-hour treatment with the NIP3 peptide. After peptide treatment, an activation phase was induced through a controlled 1-hour exposure to ATP (10 mM). Absorbance in all the experiments was analyzed using a microplate spectrophotometry system (Molecular Devices Inc., Silicon Valley, CA, USA) at the appropriate wavelengths.

To activate NLRC4 inflammasomes, cells were initially exposed to LPS (10 ng/ml; Sigma-Aldrich) for 3 h, followed by a 1-hour treatment with the synthesized peptides. Subsequently, to facilitate optimal interaction and intracellular delivery of the flagellin derivative FLA-ST (5 μg/ml; Invivogen), Lipofectamine 2000 was employed during a 6-hour incubation period. Sequentially, induction of AIM2 inflammasome activation in THP-1 cells was achieved after a differentiation regimen initiated by exposure to 80 nM PMA in a serum-free medium for 3 h. Subsequently, cells were subjected to overnight incubation in a complete medium supplemented with PMA. Upon achieving a cell density of 10^5^ cells per well, the priming phase was executed by exposing the cells to LPS (Sigma-Aldrich Co.) at a concentration of 100 ng/ml for 6 h. After this priming, the NIP3 peptide was introduced at varying concentrations of 1, 10, and 20 μM and administered for 1 h. After peptide treatment, the cells were subjected to an overnight co-stimulation with 1 μg/ml poly(deoxyadenylic-thymidylic) acid [poly(dA:dT)] in conjunction with Lipofectamine 2000, which served as the vehicle for nucleic acid delivery. This co-stimulation facilitated robust activation of the AIM2 inflammasome. Supernatants derived from the cell cultures post-co-stimulation were meticulously collected and subjected to ELISA analysis, allowing accurate quantification of the secreted IL-1β levels. To ensure the fidelity of the experimental observations, a triplicate setup was employed for each distinct experimental cohort. Furthermore, control conditions involving the treatment of cells with the vehicle were systematically integrated, thereby establishing an essential baseline for contextual interpretation.

### 4.7 Protein quantification and western blot analysis

To extract total protein, the M-PER Mammalian Protein Extraction Reagent (Thermo Fisher Scientific, Inc.) was used, and the amount of protein was determined using the bicinchoninic acid (BCA) assay (Sigma-Aldrich Co.). The western blotting procedure, which entailed gel electrophoresis and transfer, was performed using the Mini-PROTEAN Tetra Cell and Mini Trans-Blot Electrophoretic Transfer Cell System (both from Bio-Rad Laboratories, Hercules, CA, USA). The membranes were then exposed to specific primary antibodies (see below) at a concentration of 1:1000 with gentle agitation at 4 °C overnight. Antibodies targeting IL-1β and NLPR3 were obtained from Cell Signaling Technology Inc. (Danvers, MA, USA), and those against β-actin and caspase-1 were obtained from Santa Cruz Biotechnology Inc. (Dallas, TX, USA). The membranes were washed with phosphate-buffered saline containing 0.1% Tween 20 on the next day and subsequently treated with a peroxidase-conjugated anti-mouse or anti-rabbit IgG antibody at a dilution of 1:1000 for 2 h. The SuperSignal West Pico ECL solution (Thermo Fisher Scientific, Inc.) was used to detect the proteins, and imaging was performed using a ChemiDoc™ Touch Imaging System (Bio-Rad Laboratories).

### 4.8 Quantitative LDH release assay for cell death assessment

To assess cell death, a quantitative assay was employed to measure LDH release into cell supernatants. After completion of the desired incubation period, the supernatant was subjected to gentle centrifugation at 500 × *g* for 5 min at a temperature of 4 °C, ensuring the removal of any residual cells. LDH released in the cell supernatants was measured using the CytoTox 96® Non-Radioactive Cytotoxicity Assay (G1780, Promega, Madison, WI, USA) in accordance with the manufacturer's instructions. Absorbance values were recorded at 490 nm, and the obtained results were expressed as a percentage of LDH release, duly normalized to the total lysis.

### 4.9 Statistical analysis

Statistical analysis was performed by computing t-tests, and the software packages used for this purpose included SigmaPlot 12.0 (Systat Software Inc., San Jose, CA, USA) and GraphPad Prism 5 (GraphPad Software, Inc., San Diego, CA, USA). The experiments were performed independently for a minimum of four times, and statistical significance was established at P values less than 0.05 or 0.01.

## Associated content

none.

## Supporting Information

Circular dichroism (CD) spectra of NIPs (Fig. S1); Physics-derived features of NIPs (Table S1); Helical wheel projection of NIPs (Fig. S2); Correlation analysis of positively charged residues and IC_50_ of AI-designed peptide sequences (Table S2); Variations in distances between residues during interfacial interactions (Fig. S3); Cell viability assessment using MTT Assay following exposure to varied concentrations of NIP3 peptide (Fig. S4) and the model of the NLRP3 inflammasome assembly and interface interactions (Fig. S5).

## Funding

The present study received financial support from various sources, including the Korea Drug Development Fund, which is funded by the Ministry of Science and ICT, the Ministry of Trade, Industry, and Energy, and the 10.13039/100008903Ministry of Health and Welfare (HN21C1058). The 10.13039/501100001321National Research Foundation of Korea also provided financial support through the following grants: NRF-2022M3A9G1014520, 2023R1A2C2003034, 2019M3D1A1078940, and 2019R1A6A1A11051471.

## CRediT authorship contribution statement

Conceptualization, methodology, data curation, and writing-original draft preparation: B.A., A.A., and S.C.; Investigation and formal analysis: B.A., A.A., and M.F.; supervision: S.C.; writing, reviewing, editing: all authors.

## Declaration of Competing Interest

The authors declare that the research was conducted in the absence of commercial or financial relationships that could be construed as a potential conflict of interest.

## Data Availability

The peptide sequence data and code for generating the RNN LSTM model is available at https://github.com/BilalRehman01/Peptide. The manuscript includes all the remaining data.
