# Supplementary material for: Accelerated NLRP3 inflammasome-inhibitory peptide design using a recurrent neural network model and molecular dynamics simulations
Source: Comput Struct Biotechnol J. 2023 Sep 29;21:4825–35. doi: 10.1016/j.csbj.2023.09.038 (PMC10579963; doi:10.1016/j.csbj.2023.09.038)
Supplement: Figure S1 — Supplementary material [file mmc1.docx]

Supplementary Information

Accelerated NLRP3 inflammasome inhibitory peptide design using a recurrent neural network model and molecular dynamics simulations

Bilal Ahmad^1, 2*^, Asma Achek^1, 3^, Mariya Farooq^1, 2^, and Sangdun Choi^1, 2*^

^1^Department of Molecular Science and Technology, Ajou University, Suwon 16499, Korea.

^2^S&K Therapeutics, Ajou University Campus Plaza 418, Worldcup-ro 199, Yeongtong-gu, Suwon 16502, Korea.

^3^Technology Development Platform, Institut Pasteur Korea, Seongnam 13488, Korea.

^*^Email: bilalpharma77@gmail.com; sangdunchoi@ajou.ac.kr.


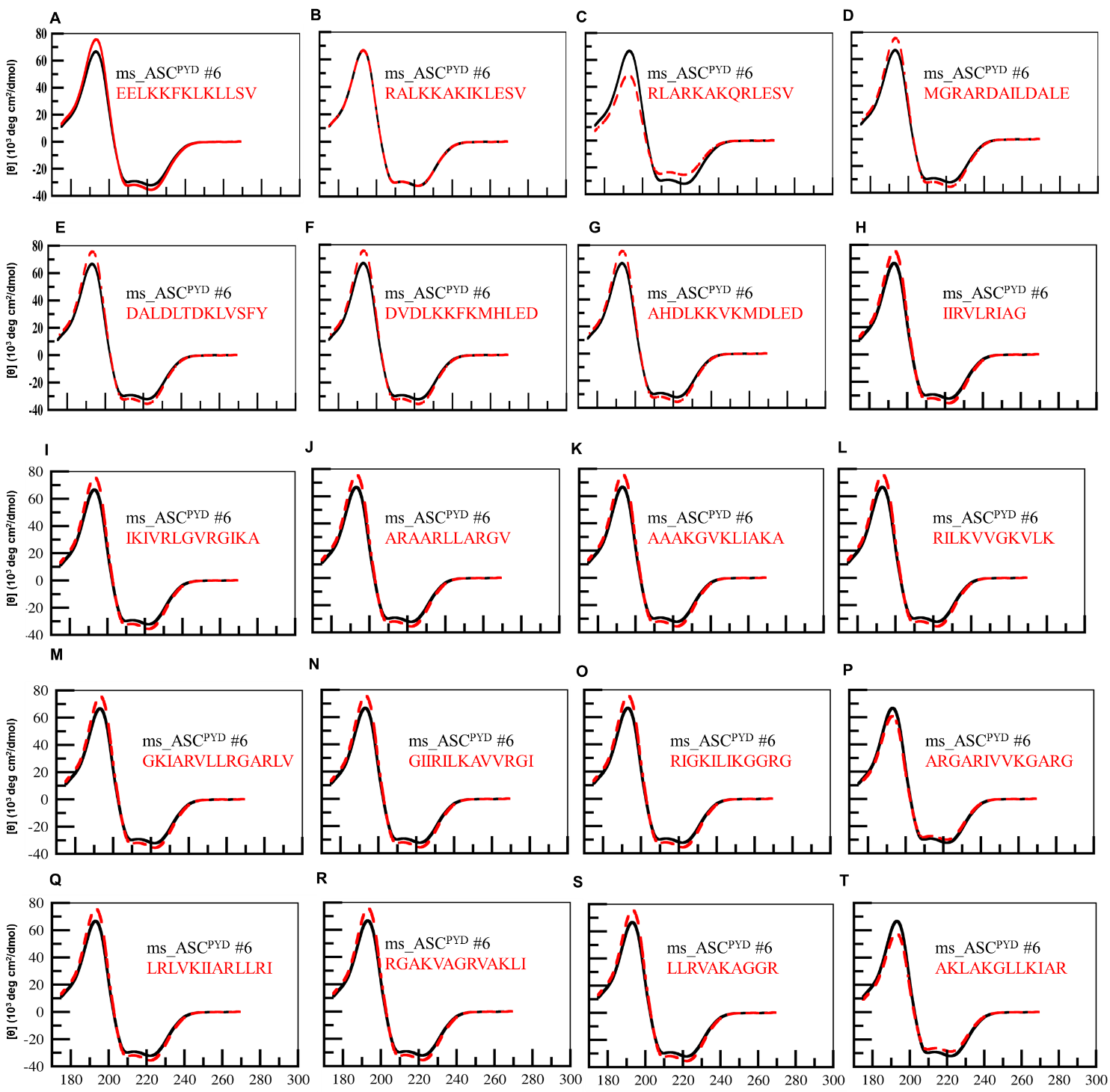


**Figure S1**. Circular dichroism (CD) spectra of NIPs. Asymmetry between msˍASCPYD#6 and generated peptides (A-T) was compared using CD spectra.

**Table S1**. Analysis of top 20 AI-designed NLRP3 inhibitory sequences based on physics-derived features.

| **Name** | **Sequence** | **Positive Residues** | **ΔG_bind_ (kcal/mol)** | | **Mean** | **Variance** |
| --- | --- | --- | --- | --- | --- | --- |
|  |  |  | **NLRP3^PYD^** | **ASC^PYD^** |  |  |
| NIP1 | EELKKFKLKLLSV | 4 | -20.34 | -29.94 | 5.90 | 1.39 |
| NIP2 | RALKKAKIKLESV | 5 | -24.45 | -45.68 | 6.52 | 1.42 |
| NIP3 | RLARKAKQRLESV | 5 | -28.38 | -48.90 | 6.87 | 1.57 |
| NIP4 | MGRARDAILDALE | 2 | -18.47 | -17.43 | 6.65 | 1.79 |
| NIP5 | DALDLTDKLVSFY | 3 | -37.49 | -24.34 | 6.45 | 1.27 |
| NIP6 | DVDLKKFKMHLED | 3 | -32.46 | -20.23 | 7.46 | 1.35 |
| NIP7 | AHDLKKVKMDLED | 3 | -29.63 | -18.75 | 8.28 | 1.80 |
| NIP8 | IIRVLRIAG | 2 | -19.32 | -20.54 | 6.29 | 1.71 |
| NIP9 | IKIVRLGVRGIKA | 4 | -23.67 | -24.43 | 6.52 | 1.24 |
| NIP10 | ARAARLLARGV | 3 | -25.21 | -24.76 | 7.16 | 1.19 |
| NIP11 | AAAKGVKLIAKA | 3 | -28.78 | -29.32 | 7.20 | 1.39 |
| NIP12 | RILKVVGKVLK | 4 | -31.53 | -33.67 | 6.52 | 1.24 |
| NIP13 | GKIARVLLRGARLV | 4 | -34.23 | -30.43 | 6.41 | 1.28 |
| NIP14 | GIIRILKAVVRGI | 3 | -31.32 | -26.34 | 7.62 | 1.46 |
| NIP15 | RIGKILIKGGRG | 4 | -27.43 | -28.30 | 6.64 | 1.76 |
| NIP16 | ARGARIVVKGARG | 4 | -25.54 | -33.45 | 6.40 | 1.26 |
| NIP17 | LRLVKIIARLLRI | 4 | -27.45 | -34.43 | 6.45 | 1.29 |
| NIP18 | RGAKVAGRVAKLI | 4 | -31.23 | -35.05 | 8.37 | 1.50 |
| NIP19 | LLRVAKAGGR | 3 | -24.09 | -28.79 | 7.62 | 1.46 |
| NIP20 | AKLAKGLLKIAR | 4 | -29.76 | -27.54 | 7.20 | 1.39 |

Physics-derived features such as mean and variance of the number of contacts between positive amino acids and PYDs. The binding affinity for the top 20 AI-generated sequences were calculated using MMPBSA method by extract the frames from MD trajectories.


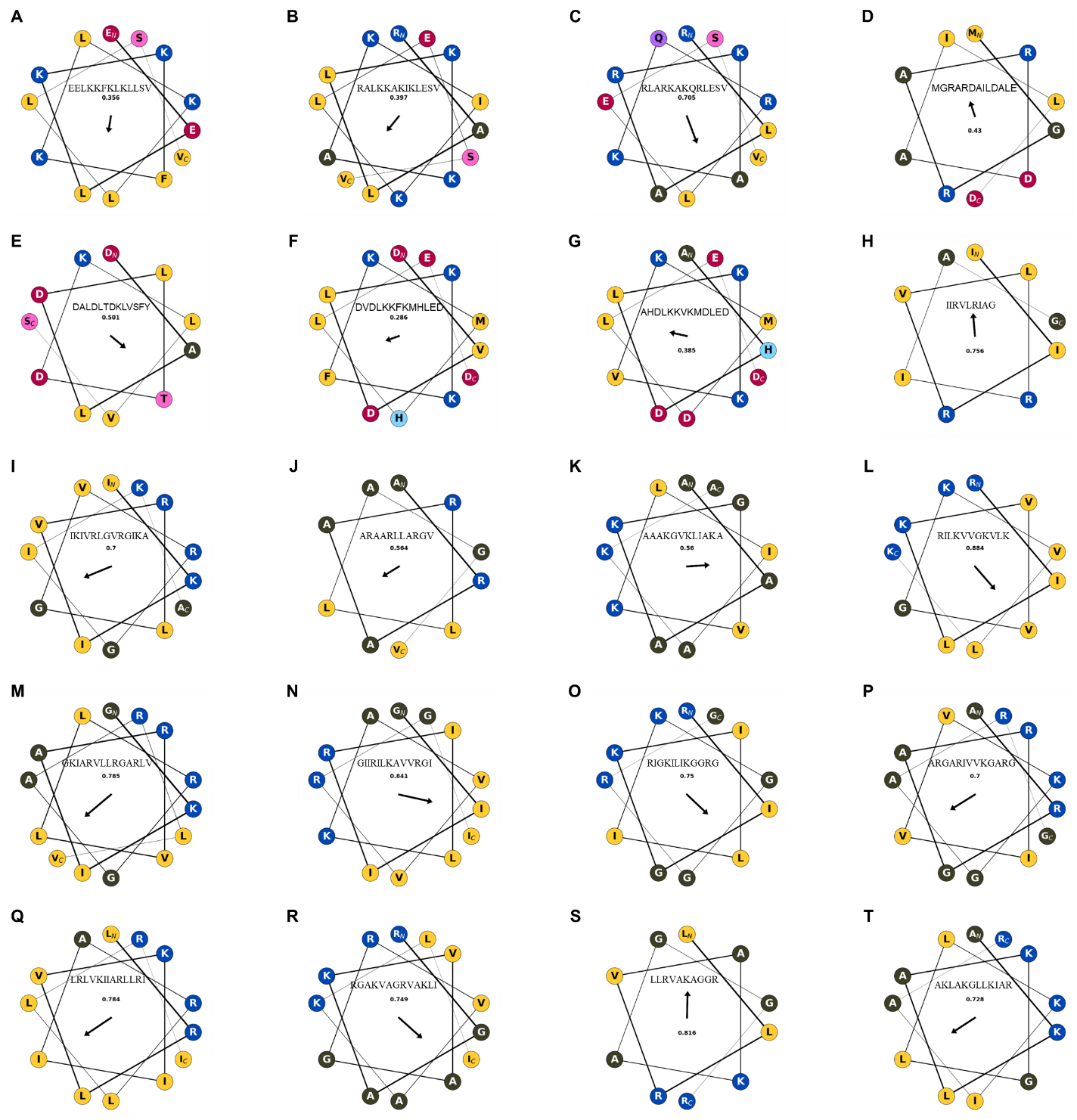


**Figure S2.** Helical wheel projection of NIPs. Positively charged, negatively charged, hydrophobic, and other residues are highlighted in blue, red, yellow, and black, respectively. Eisenberg’s hydrophobic moment for the idealized α-helical conformation is indicated by the arrow along with the corresponding value.

**Table S2.** Correlation analysis of positively charged residues and IC_50_ of AI-designed peptide sequences.

| **Name** | **Sequence** | **Positive Residues** | **IC_50_(µM)** |
| --- | --- | --- | --- |
| NIP1 | EELKKFKLKLLSV | 4 | >50 |
| NIP2 | RALKKAKIKLESV | 5 | 10 |
| NIP3 | RLARKAKQRLESV | 5 | 2 |
| NIP4 | MGRARDAILDALE | 2 | >50 |
| NIP5 | DALDLTDKLVSFY | 3 | 20 |
| NIP6 | DVDLKKFKMHLED | 3 | >100 |
| NIP7 | AHDLKKVKMDLED | 3 | >50 |


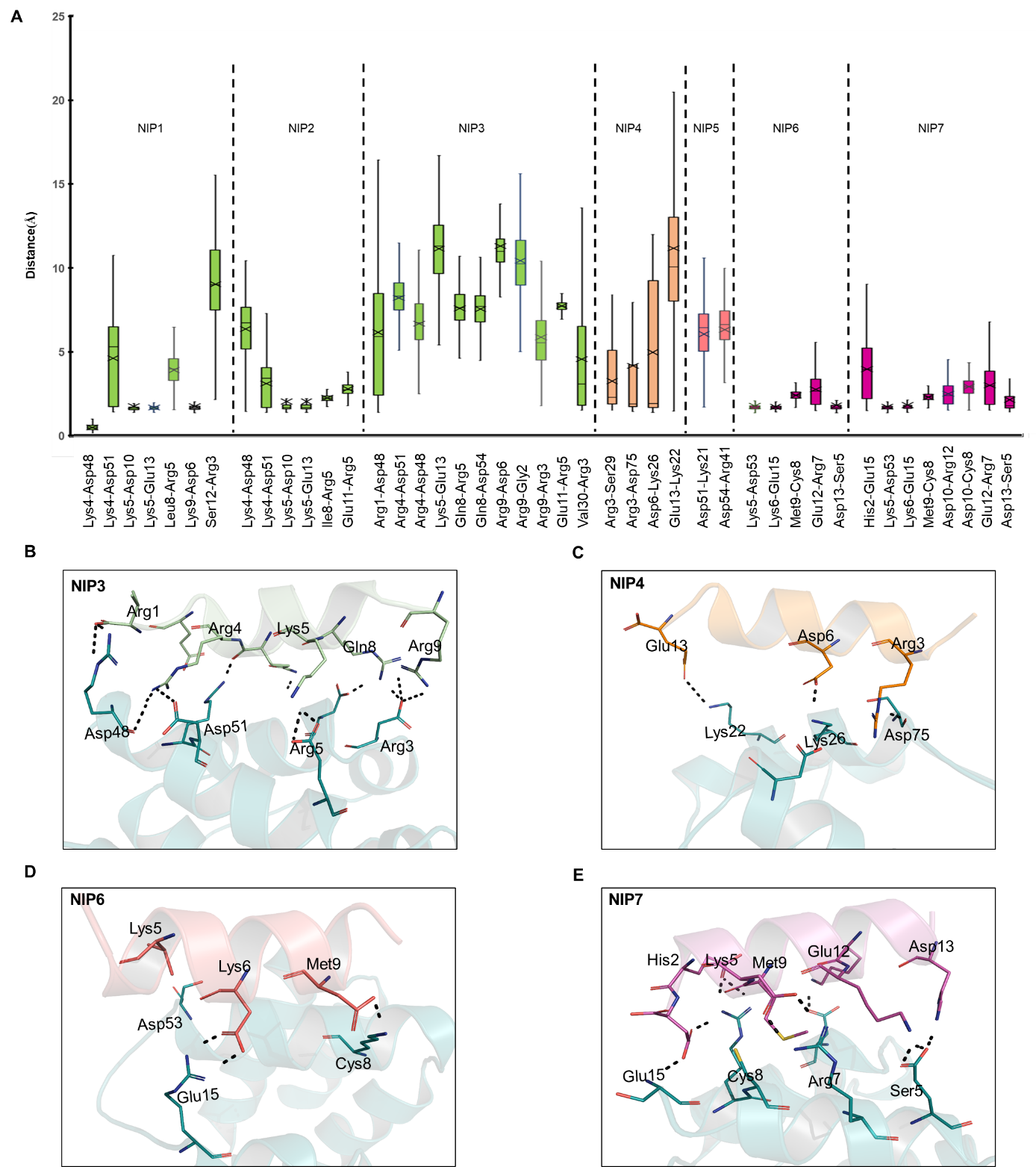


**Figure S3.** Variations in distances between residues during interfacial interactions. Computed averages and fluctuations of interatomic distances for selected residues. The structural features of the crucial interface interactions between the selected peptides (B)NIP3, (C) NIP4, (D) NIP6 and (E) NIP7, and the PYD were depicted through the analysis of the first frame from MD trajectories.
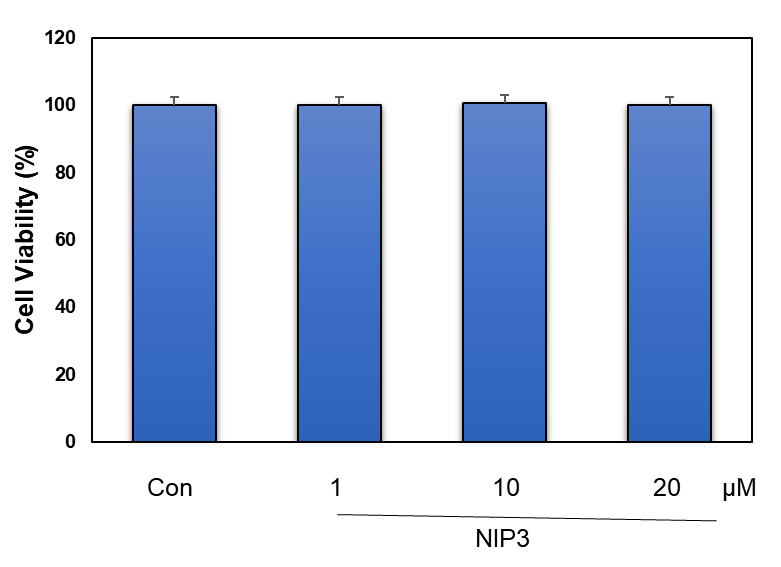


**Figure S4.** Cell viability assessment using MTT assay following exposure to varied concentrations of NIP3 peptide.


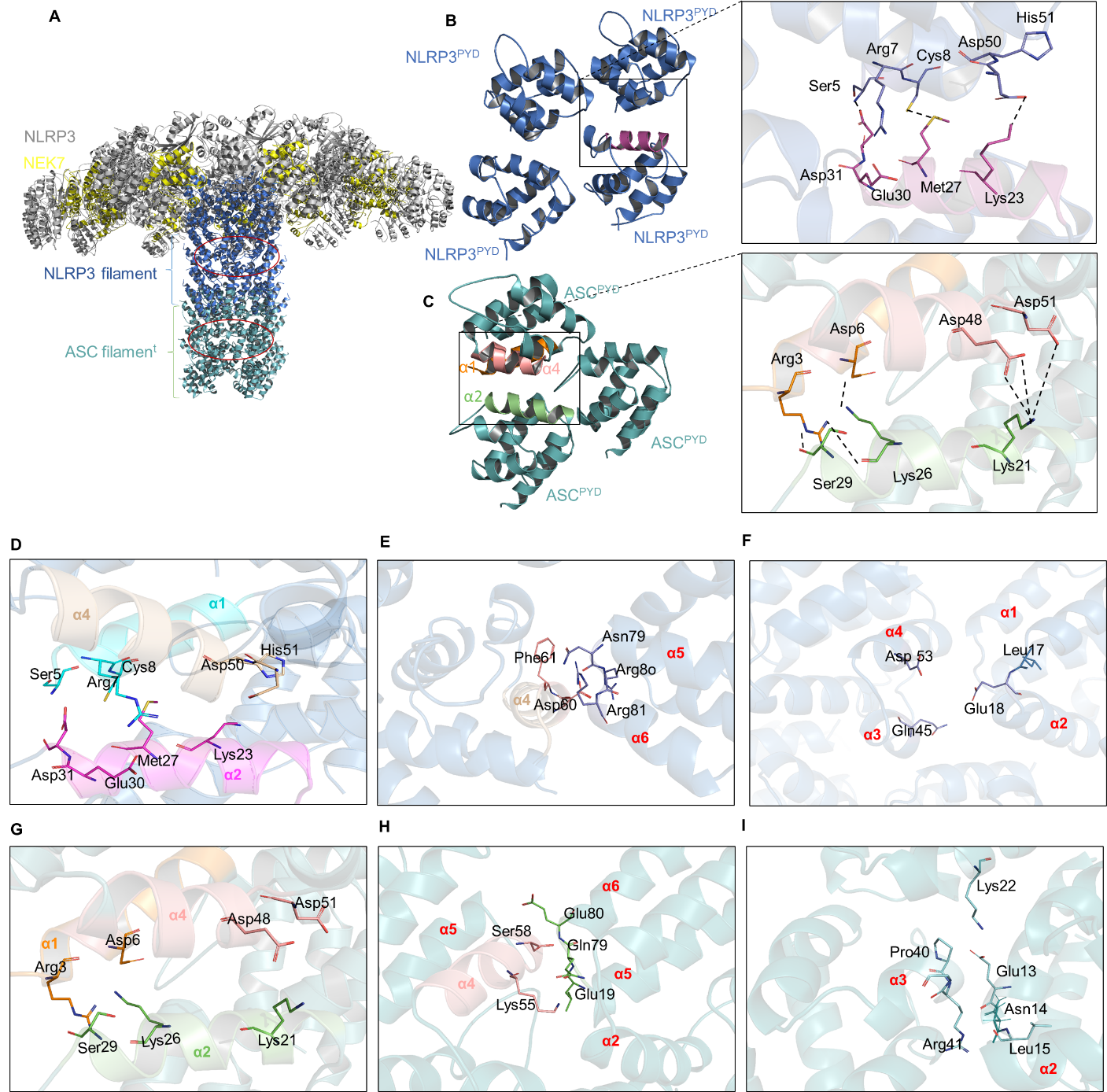


**Figure S5.** The model of the NLRP3 inflammasome assembly and interface interactions. (A) A model of the NLRP3 inflammasome assembly. Upstream sensing protein NLRP3 oligomerizes upon activation to form a platform of PYDs that induces ASC filament assembly through PYD–PYD interactions. ASC^PYD^ self-associates and interacts with NLRP3^PYD^ through equivalent protein regions. (B) The interacting helices of NLRP3^PYD^ (α1:cyan, α2:magenta, α 4:wheat)and (C) ASC^PYD^ (α1:organge, α2:limegreen and α4:brick red)were used to search motif from different databases for the long-short-term memory (LSTM) network model training. Type I, II, and III interface interactions of (D–F) NLRP3^PYD^ and (G–I) ASC^PYD^ in detail. The interface interacting motifs are highlighted
